# Salt-Induced Transformations of Hybrid Micelles Formed by Anionic Surfactant and Poly(4-vinylpyridine)

**DOI:** 10.3390/polym14235086

**Published:** 2022-11-23

**Authors:** Alexander L. Kwiatkowski, Vyacheslav S. Molchanov, Alexander I. Kuklin, Yuri M. Chesnokov, Olga E. Philippova

**Affiliations:** 1 Physics Department, Moscow State University, 119991 Moscow, Russia; 2 Joint Institute for Nuclear Research, 141980 Dubna, Russia; 3 National Research Center, Kurchatov Institute, 123182 Moscow, Russia

**Keywords:** hybrid micelles, polymer-surfactant complexes, anionic surfactant, hydrophobic polymer

## Abstract

Salt-induced structural transformation of charged hybrid surfactant/polymer micelles formed by potassium oleate and poly(4-vinylpyridine) was investigated by cryo-TEM, SANS with contrast variation, DLS, and 2D NOESY. Cryo-TEM data show, that at small salt concentration beads-on-string aggregates on polymer chains are formed. KCl induces the transformation of those aggregates into rods, which is due to the screening of the electrostatic repulsion between similarly charged beads by added salt. In a certain range of salt concentration, the beads-on-string aggregates coexist with the rodlike ones. In the presence of polymer, the sphere-to-rod transition occurs at higher salt concentration than in pure surfactant system indicating that hydrophobic polymer favors the spherical packing of potassium oleate molecules. The size of micelles was estimated by DLS. The rods that are formed in the hybrid system are much shorter than those in polymer-free surfactant solution suggesting the stabilization of the semi-spherical endcaps of the rods by embedded polymer. 2D NOESY data evidence that in the spherical aggregates the polymer penetrates deep into the core, whereas in tighter packed rodlike aggregates it is located mainly at core/corona interface. According to SANS with contrast variation, inside the rodlike aggregates the polymer adopts more compact coil conformation than in the beads-on-string aggregates. Such adaptive self-assembled polymer-surfactant nanoparticles with water-insoluble polymer are very promising for various applications including drag reduction at transportation of fluids.

## 1. Introduction

Amphiphilic molecules of surfactants in aqueous solutions are able to form molecular self-assemblies called micelles [1,2,3,4]. In the case of ionic surfactants, the micelle formation is governed by a balance between the attraction of hydrophobic tails, the free energy of formation of the micellar core-solvent interface and the electrostatic repulsion of the hydrophilic heads of the surfactant molecules [3,4]. Surfactant micelles are of particular scientific interest, since they can be employed in a variety of applications, such as detergents [5], thickeners [6,7,8], drag reducing agents [9] or drug delivery systems [10,11]. Within the micelles the surfactant molecules are bound together by weak hydrophobic interactions that makes them sensitive to external conditions [7,8,12]. In particular, the addition of salt to the solution of ionic surfactant transforms the spherical micelles into the rodlike ones [13,14,15,16,17,18]. Transformation occurs due to partial screening of electrostatic repulsion of the similarly charged head groups of the surfactant by oppositely charged ions of added salt that favors tighter packing of surfactant ions.

The rodlike micelles of surfactants are widely used as drag reducing additives to diminish the resistance loss in the conveying flow, while petroleum, natural gas and water are transported in the pipeline networks [9,19]. Non-covalent binding of surfactant molecules in rodlike micelles provides their anti-degradation ability at high temperatures and strong shear flows, in contrast to high-molecular-weight polymers that undergo irreversible degradation [19,20]. However, the drag reducing effect of rodlike micelles is much lower [19] than that of the polymers. A promising way to modify the structure and properties of surfactant drag reducing agents is to combine them with polymeric ones [19]. For this aim, several polymer-surfactant complexes could be applied [21,22,23,24]. In addition to drag reduction, many industrial processes rely on tuning the properties by an appropriate blending of polymer and surfactant [23]. It was observed that in the polymer-surfactant complexes, rodlike micelles acquire unique properties commonly found in polymers, e.g., toughness [24,25] or flexibility [26]. Moreover, some synergistic effects can be observed. For instance, complex of poly(ethylene glycol) (PEG) and sodium dodecyl sulfate (SDS) demonstrates higher ability to solubilize hydrophobic agents as compared to free SDS micelles [11].

By now, mainly complexes of ionic surfactants with charged and uncharged water-soluble polymers were studied [11,25,26,27,28,29,30,31,32,33,34,35]. In oppositely charged polymer/surfactant systems, the electrostatic interactions between the charges on the respective components dominate, whereas in the uncharged polymers/ionic surfactants systems the hydrophobic interactions are mainly involved in the aggregation [36,37]. Complexes with uncharged water-soluble polymers are usually considered to have a necklace-like (beads-on-string) structure [11,25,26,27,28,29,30], where the spherical micelles of the surfactant with embedded polymer (beads) are slightly connected to each other by short polymer strings. Within the beads, the hydrophobic polymer segments partially penetrate and wrap around the polar head group region of the surfactant micelles [38].

As to the complexation of ionic surfactants with oppositely charged polyelectrolytes, it can give a variety of structures including beads-on-string aggregates [27,28,39,40,41] and hybrid wormlike micelles (WLMs) with embedded polymer chains [31,33,34,35]. In the last case, the conformation of the polyelectrolyte in the aggregate is analogous to that of a polymer chain confined in a “tube” or cylindrical pore in which the pore walls are attractive [42]. The formation of complexes of ionic surfactants with oppositely charged polyelectrolytes is governed by three effects: electrostatic and hydrophobic surfactant-polymer interactions and translational entropy of the counterions [36,39].

An important factor affecting the structure and properties of polymer-surfactant complexes is the concentration of salt in the solution. In the case of the complexation of ionic surfactants with oppositely charged polyelectrolytes salt hinders the complex formation, since it weakens the electrostatic attraction between the components and decreases the gain in translational entropy of counterions released from the polyelectrolyte [43].

In the case of complexes of ionic surfactants with uncharged water-soluble polymers the addition of salt can affect the structure of the complexes and the characteristic concentrations of the complex formation [44]. For example, the concentration at which the surfactant molecules start to form micelles on the polymer (critical aggregation concentration, CAC) for SDS interacting with poly(ethylene oxide) (PEO) decreases significantly upon addition of salt [30], indicating stronger binding of the surfactant molecules to the polymer chain caused by the screening of the repulsion of the charged heads.

Much less is known about the effect of salt on the complexes, containing uncharged water-insoluble polymer. On an example of poly(4-vinylpyridine) (P4VP)/potassium oleate complexes it was shown that in the absence of salt they represent beads-on-string structures [45], whereas in the excess of salt (800 mM KCl) they form polymer-loaded WLMs [46]. However, the transition between those two types of complexes with increasing salt concentration has not been studied. Also, it is important to compare the structural transformations of polymer-surfactant complexes with those in the polymer-free micelles, since it will allow to reveal the contribution of polymer to this process. Combination of surfactant micelles with water-insoluble polymer leads to the formation of hybrid micelles, which can be more stable than the polymer-free micelles. Such hybrid micelles represent promising building blocks for the construction of various supramolecular architectures [34]. The dynamic nature of the non-covalent interactions exploited in the design of such systems will result in a production of smart functional material adaptive to the environment [34]. Also, such hybrid micelles are quite promising as modern additives for drug reduction, since they are more stable than the polymer-free counterparts while retaining the anti-degradation properties.

The present paper is aimed at the investigation of the structural transformations of the complexes of uncharged water-insoluble polymer P4VP with ionic surfactant potassium oleate that were induced by the addition of inorganic salt KCl. Only complexes saturated with polymer were studied. The studies were performed by several complementary techniques: SANS with contrast variation to reveal the structure of the aggregates as a whole and of their components, cryo-TEM to visualize the aggregates, DLS to estimate the size of micelles, 2D NOESY spectroscopy to determine the location of polymer in the complex. The differences in the structure evolution of polymer-surfactant complexes upon addition of salt in comparison with the polymer-free micelles were revealed, which permitted to identify the effect of polymer in this process. The revealed structural transitions offer useful information for designing new polymer/surfactant materials for nanotechnological and drag reduction [19] applications.

## 2. Materials and Methods

### 2.1. Materials

Anionic surfactant potassium oleate (>98% purity, TCI Europe, Zwijndrecht, Belgium); hydrophobic polymer P4VP (molecular mass of 60,000 g/mol, contour length of 183 nm, Merck, Burlington, VT, USA), inorganic salt potassium chloride (>99.5% purity, Fluka, Buchs, Switzerland) were used as received. Ethanol (>99% purity, Sigma-Aldrich, Burlington, VT, USA) was employed for preliminary solubilization of P4VP. Water purified with Millipore Milli-Q system was used as a solvent. For SANS and 2D NOESY experiments, deuterium oxide (99.9% purity, Sigma-Aldrich, Burlington, USA) was taken instead of water.

### 2.2. Preparation of Samples

Aqueous solutions of polymer-free micelles of surfactant were prepared by mixing appropriate amounts of potassium oleate and potassium chloride with purified water. Concentration of potassium oleate was fixed at 0.094 M. The concentrations of KCl were equal to 67, 110 and 160 mM. The pH of the solutions was adjusted to 11 with KOH. Solutions of polymer-free micelles were stirred for 1 day by magnetic stirrer.

For preparation of the solutions of polymer-surfactant complexes a stock solution of P4VP in ethanol (5 wt%) was used. Appropriate amount of the stock solution was added to a vial and left at room temperature. After full evaporation of ethanol, a polymer film was formed at the bottom of the vial. To prepare the solutions of polymer-surfactant complexes in the presence of different concentrations of KCl, the corresponding solutions of polymer-free micelles were poured into the vial containing the polymer film. Then the solutions were stirred for 1 day to accelerate the solubilization of P4VP into the micelles.

Note that maximum amount of polymer solubilized in the micelles depended on the concentration of salt in the solutions. The maximum content of P4VP in the complexes was determined in the following way. The concentration of P4VP was gradually increased until the appearance of a precipitate. The largest concentration of P4VP in the solution without precipitate was considered as the concentration of polymer in the saturated polymer-surfactant complex. For 67, 110 and 160 mM KCl solutions, this concentration was equal to 0.14, 0.12 and 0.08 mol of monomer units of P4VP/L (monomol/L), respectively. Further experiments were carried out with the complexes saturated with P4VP.

### 2.3. Cryogenic Transmission Electron Microscopy (Cryo-TEM)

Cryo-TEM specimens in the native conditions were prepared with the Vitrobot (FEI) [47] by applying the sample onto a Lacey TEM grid (Ted Pella). The excess of solution was blotted with filter paper at T = 20 °C, after that the grid was immediately plunged into liquid ethane. 

Cryo-TEM study was conducted using Titan Krios (ThermoFisher Scientific) equipped with Falcon 2 direct electron detector (ThermoFisher Scientific) and Image Corrector (CEOS, Germany) operated at 300 kV. Images were obtained using EPU software (ThermoFisher Scientific) at 37,000× magnification with 1.72 Å pixel size, defocus range 2–4 µm and total dose ~50 e/Å^2^. To obtain deconvolved and denoised images Warp software [48] was applied.

### 2.4. Small-Angle Neutron Scattering (SANS)

SANS data was obtained at the YuMO instrument [49] (Frank Laboratory of Neutron Physics, Joint Institute for Nuclear Research, Dubna, Russia) at the high-flux pulsed reactor IBR-2M. The details of the experiment are reported elsewhere [50]. The following scattering length densities of the compounds of polymer-surfactant mixtures were used: 6.38 × 10^−6^ Å^−2^ for D_2_O, −0.56 × 10^−6^ Å^−2^ for H_2_O, 0.15 × 10^−6^ Å^−2^ for potassium oleate and 2 × 10^−6^ Å^−2^ for P4VP. The contrast matching technique was applied to get scattering curves separately from potassium oleate molecules and from P4VP chains in the polymer-surfactant complexes. To match the scattering from P4VP the mixture containing 37 vol.% of D_2_O and 63 vol.% of H_2_O was used as a solvent. The mixture of 90 vol.% of D_2_O and 10 vol.% of H_2_O was used for matching the scattering from molecules of potassium oleate. The temperature was kept at 20 °C.

### 2.5. Dynamic Light Scattering (DLS)

The DLS measurements were carried out on an ALV/DLS/SLS-5022F goniometer equipped with a stepping-motor-driven variable-angle detection system and ALV6010/EPP digital correlator. Helium-neon laser with a wavelength of 632.8 nm was used as a light source. The temperature was kept at 20 °C by a Lauda Ecoline RE 306 system. Before the measurements the solutions were filtered through 0.45 μm Millipore Millex-FG filter. Details of the experiments and data treatment are described elsewhere [51].

### 2.6. Two-Dimensional 1H Overhauser NMR-Spectroscopy

2D NOESY experiments were carried out on a Bruker AV-600 spectrometer at a proton resonance frequency equal to 600 MHz, using a mixing time of 800 ms and the relaxation delay of 2 s between the scans. Data were acquired for 10 h. The proton chemical shifts were referenced to the D_2_O signal at 22 °C equal to 4.70 ppm.

## 3. Results and Discussion

### 3.1. Structural Transitions of Polymer-Free Micelles upon Addition of Salt

**Figure 1:**
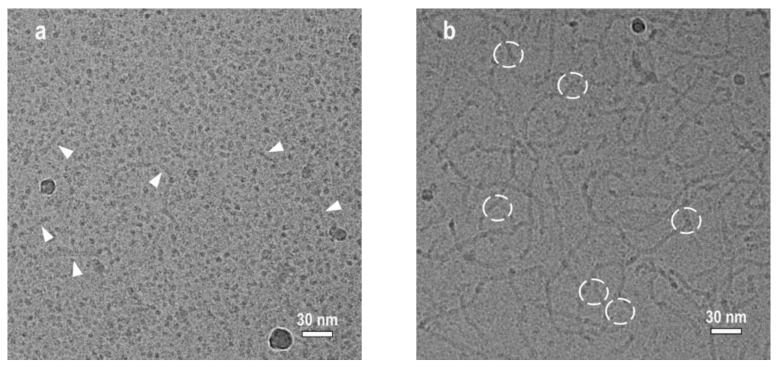
Cryo-TEM images of 94 mM aqueous solutions of potassium oleate in the presence of 67 mM (**a**) and 160 mM (**b**) KCl at pH = 11. Arrows point to rodlike micelles in 67 mM KCl solution. Circles indicate to branching points of WLMs in 160 mM KCl solution.

In Figure 1 the cryo-TEM micrographs of 94 mM solutions of potassium oleate with added 67 and 160 mM of KCl are depicted. At low salt content spherical micelles are mostly present in the solution (Figure 1a). However, a few rodlike micelles with the length from 30 to 100 nm can also be observed. Similar cryo-TEM images of potassium oleate solutions with low salt content were previously obtained by C. Flood et al. [13] According to literature [13,14,15,16,17,18], short rodlike micelles are formed from spherical ones, due to partial screening of electrostatic repulsion of the similarly charged head-groups of potassium oleate on the micellar surface by salt ions.

At higher salt content (160 mM KCl), the cryo-TEM image becomes quite different (Figure 1b). At these conditions, long WLMs are observed. Most of them are branched. Note that in cryo-TEM images branches have to be carefully distinguished from the entanglements and overlaps of WLMs lying in different layers [52,53,54]. Since marked junctions are clearly determined to be “Y-shaped” and do not appear darker than the surrounding media, they are considered to be branching points. The formation of branches in WLMs of ionic surfactants is usually observed at high content of salts [7,13,16,53,55]. Enhanced screening of the electrostatic repulsion of the charged head groups of the surfactant molecules by salt fosters their tighter packing so that the spherical endcaps become unfavorable and some of them are eliminated by forming branching points [9,13,55]. In the case of potassium oleate, the branched micelles were previously detected in 800 mM KCl [13]. In the present study, high resolution cryo-TEM picture shows many branching points on WLMs already at as low salt concentration as 160 mM (Figure 1b). Several spherical objects can also be found in Figure 1b. But they are darker than the background or/and localized at the ends and cross-points of the WLMs. These could be artifacts resulting from the interaction of electrons with bends and intersects of WLMs lying on different depths of the sample [54].

**Figure 2:**
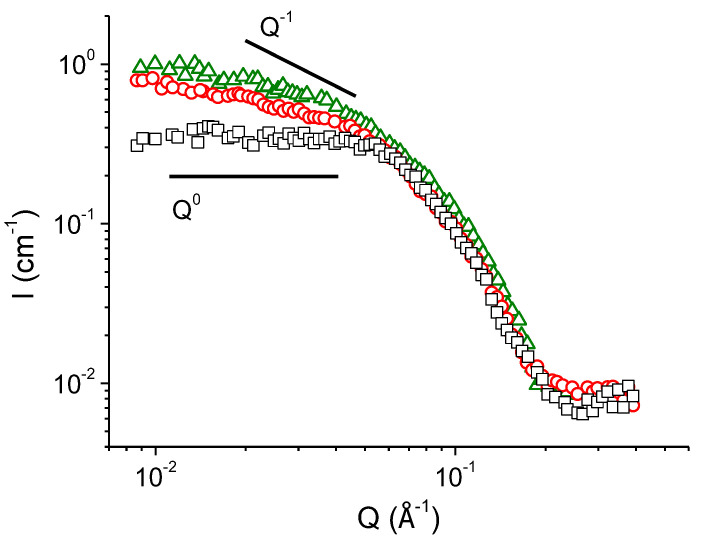
SANS curves of 94 mM solutions of potassium oleate in the solvent containing 37 vol% D_2_O and 73 vol% H_2_O at pH 11 in the presence of different concentrations of KCl: 67 mM (squares), 110 mM (circles) and 160 mM (triangles). Solid lines show slopes of the *Q*^0^ and *Q*^−1^ dependences.

SANS curves of the polymer-free micellar solutions with different concentrations of added KCl are demonstrated in Figure 2. The curve, corresponding to 67 mM KCl solution, reaches plateau in the low- and medium-*Q* regions. Such behaviour is typical to the solutions of ionic surfactants containing spherical micelles [15,45,56,57,58,59]. Frequently, a pronounced structure peak associated with Coulomb repulsion of the surfactant headgroups is present in the low-*Q* region of the curves [45]. However, it is not observed in the present case, suggesting relatively high screening of the electrostatic interactions of surfactant ions by salt [13,16,59]. The screening is effective enough to induce the formation of rodlike micelles from part of spherical ones, but not too strong to induce their sufficient growth in length (Figure 1a).

From Figure 2 one can see that with enhanced screening (at increasing KCl content) the slope of the SANS curves *I(Q)* in the medium-*Q* region changes from 0 to −1 that is inherent to the scattering from WLMs [58,59,60,61,62,63]. This observation is consistent with the cryo-TEM results (Figure 1), demonstrating the formation of WLMs at 160 mM KCl. Figure 2 shows that the scattering intensity at low-*Q* values increases with increasing concentration of KCl from 110 to 160 mM. It may be related to the growth of WLMs in length [16], since at higher salt content their tighter packed cylindrical central parts become more favorable as compared with the semi-spherical end-caps. In the high-*Q* region, the scattering curves perfectly overlap (Figure 2), indicating that the local micellar structure remains unchanged. Similar evolution of SANS curves was previously observed for salt-induced sphere-to-rod transition of micelles in several surfactant/salt solutions [13,16].

Therefore, the data obtained by two complementary techniques (cryo-TEM and SANS) indicate that in polymer-free micellar solutions of potassium oleate increasing salt concentration induces the transition of spherical to rodlike micelles and then to branched worms. Now let us consider how the hydrophobic polymer P4VP solubilized in the micelles affects the structural transformations of the micelles triggered by added salt.

### 3.2. Structural Transitions of Hybrid Micelles of Polymer-Surfactant Complexes upon Addition of Salt

**Figure 3:**
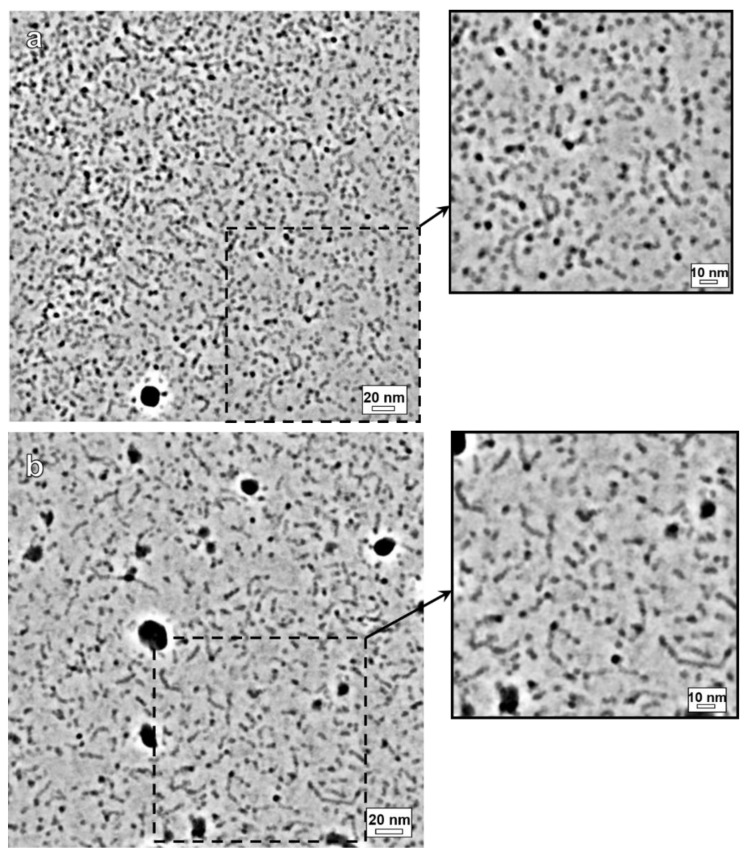
Cryo-TEM images of aqueous solutions of polymer-surfactant complexes of potassium oleate, saturated with P4VP, in the presence of different concentrations of KCl at pH 11: 67 mM (**a**) and 160 mM (**b**). The insets are maximized images of the thin layer of the specimen.

Figure 3a demonstrates a visualization of P4VP/potassium oleate polymer-surfactant complexes in 67 mM KCl obtained by cryo-TEM with noise suppression. Separated groups of several spherical micelles prevail in the image. They represent beads-on-string structures similar to those observed in salt-free solutions [45]. Such structures consist of the polymer-loaded spherical micelles (beads) of surfactant connected by thin strings of polymer. Strong electrostatic repulsion between the similarly charged beads prevents their aggregation with each other [45]. The incorporation of P4VP into the micelles of potassium oleate reduces the unfavorable contact of hydrophobic tails of surfactant with water and provides a suitable environment for water-insoluble polymer. Thus, the formation of polymer-surfactant complexes is thermodynamically favorable for both components of the system.

At low salt concentration (67 mM KCl), the structure of polymer-surfactant complexes (beads-on-string) differs essentially from that of the polymer-free surfactant aggregates (spheres and short rods) despite the same potassium oleate content in both systems. This can be explained by the fact that at weak screening of the repulsion between the charged headgroups by salt the incorporation of polymer inside the micelles pushes the surfactant heads farther from each other thereby favoring the spherical geometry of micelles. Since a single bead cannot accommodate the whole macromolecule the beads-on-string structure appears where several beads are formed on the same polymer chain.

The value of hydrodynamic radius of the beads obtained by DLS (see Supplementary Materials) coincides within the experimental error with that measured for spherical micelles in the absence of polymer, and is close to the length of the hydrophobic tail of potassium oleate [64]. This result confirms the similarity of the observed beads in the beads-on-string polymer-surfactant complexes (Figure 3a) with the spherical micelles of potassium oleate (Figure 1a).

At higher salt concentration (160 mM KCl), in polymer-surfactant system, the beads-on-string structures coexist with hybrid rodlike micelles of different lengths (Figure 3b). To the best of our knowledge the coexistence of two types of polymer-surfactant complexes is demonstrated here for the first time. Note that at 160 mM KCl, the structure of polymer-surfactant complexes (beads-on-string coexisting with rods) differs essentially from that of the polymer-free surfactant aggregates (long branched WLMs). This result indicates that polymer “stabilizes” the spherical form of the micelles (in beads-on-string structures), shortens the WLMs and eliminates their branching.

In polymer-free solution, the coexistence of spherical and rodlike micelles is also observed, but at much lower salt concentration (67 mM KCl) than in the polymer-surfactant system. So, P4VP shifts the sphere-to-rod transition of potassium oleate micelles towards higher salinity. It could be explained by the stabilization of the spherical micelles by embedded polymer.

At very high salt concentration (800 mM KCl) in P4VP/potassium oleate system, only short hybrid rodlike micelles with wide size distribution are observed [46]. Most probably, the hybrid rodlike micelles are formed from the beads-on-string structures, when the electrostatic repulsion between the beads becomes sufficiently screened by salt. Note that the observed P4VP/potassium oleate rodlike micelles in 800 mM KCl are much shorter than the polymer-free micelles at the same surfactant concentration [46]. The shortening of WLMs after addition of P4VP is due to enhanced probability of breaking of polymer-loaded WLMs at the “weak spots”, i.e., the boundaries between the polymer-containing and polymer-free sections of WLMs, where hydrophobic P4VP might contribute to the stabilization of the micellar end-caps [46,65].

**Figure 4:**
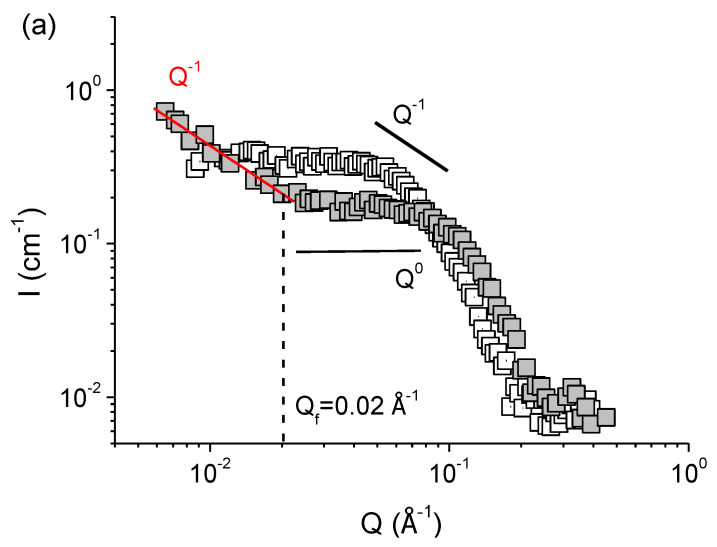

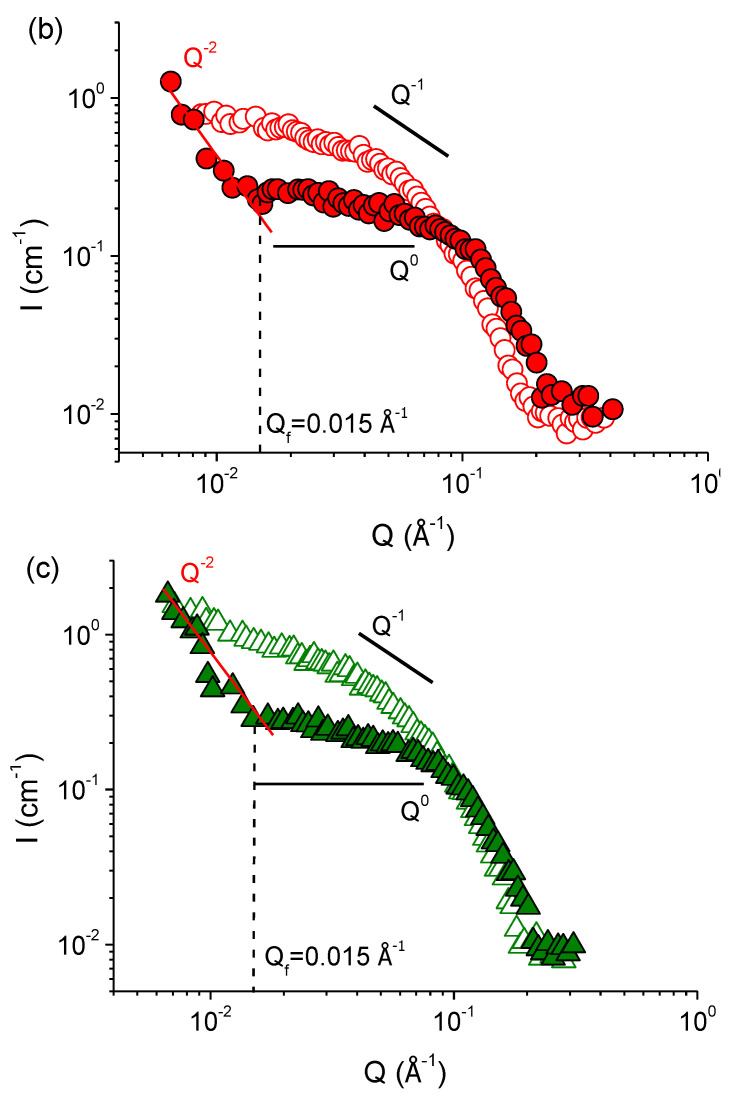
SANS curves of polymer-free solutions of potassium oleate (open symbols) and of saturated P4VP/potassium oleate complexes (filled symbols) in the presence of different concentrations of KCl: 67 mM (**a**), 110 mM (**b**) and 160 mM (**c**). Solvent (37 vol% D_2_O/63 vol% H_2_O at pH 11) matches the scattering from polymer. Black solid lines show slopes of the *Q*^0^ and *Q*^−1^ dependences. Red solid line shows the slope of the curves in the low-*Q* range. *Q_f_* corresponds to the transition between high-*Q* and medium-*Q* scaling.

SANS profiles of polymer-surfactant complexes at different concentrations of KCl, obtained in the solvent matching the scattering from P4VP (37 vol.% H_2_O/63 vol.% D_2_O), are demonstrated in Figure 4. Those curves display the scattering from the surfactant in the complexes. In the medium- and high-*Q* ranges the scattering curves *I(Q)* behave similarly to those of polymer-free micelles (Figure 2). In medium-*Q* region, the slope of the curves changes from 0 to −1 with increasing concentration of KCl from 67 to 160 mM. It indicates a gradual transformation of spherical scattering objects into rodlike ones [58,59,60,61,62,63]. Hence, current SANS data confirms the transition of beads-on-string complexes with spherical packing of surfactant molecules into the hybrid rodlike micelles. It is consistent with cryo-TEM results (Figure 3).

Whereas at all studied concentrations of salt in the absence of polymer the SANS curves have an almost constant slope in a wide range of scattering vectors 0.008 Å^−1^ < *Q* < 0.4 Å^−1^, a sharp decline is demonstrated by the curves of the polymer-surfactant complexes at *Q* < 0.015 Å^−1^ (Figure 4). A pronounced power-law dependence in the low-*Q* region points out to the formation of the mass-fractal structure upon incorporation of polymer in the micelles [66,67,68,69]. According to literature, the dependence of scattering intensity *I* of mass-fractal object of dimension *d_f_* on the scattering vector *Q* is described by the following law [66,70]:

(1) $$I(Q)=B_{f}Q^{-d_{f}},\;1\leq d_{f}\leq 3$$

where *B_f_* is the power-law prefactor. Values of *d_f_*, equal to 1 and 2, correspond to the scattering from rigid rods and Gaussian chains, respectively [60,68,71].

Expression (1) was used to obtain the fractal dimensions *d_f_* of the polymer-surfactant complexes. Values of *d_f_* at different salt concentrations are summarized in Table 1. In 67 mM KCl, *d_f_* equals to 1. After gradual increase of salt content to 110 and 160 mM it turns to ca. 2. Characteristic scale *ξ_f_* of the mass-fractal scattering could be evaluated from the intersection of the high-*Q* and medium-*Q* power-law regimes *Q_f_* in Figure 4 as *ξ_f_* = 2*π/Q_f_*. It equals to ca.40 nm (Table 1). Therefore, the fractal dimension allows judging about the morphology of the polymer-surfactant complex at a large scale comparable to the size of the whole macromolecule. At low salt content, at such a large scale the beads-on-string complexes are aligned like rigid rods, which can be due to the electrostatic repulsion between similarly charged beads. At higher salt concentrations (110–160 mM KCl), the screening of electrostatic repulsion permits to the complexes to adopt Gaussian coil conformation. Similar effect of screening on the fractal dimension of complexes of ionic surfactants by polyelectrolytes was previously demonstrated in the literature [72,73,74,75], in particular, for complexes bovine serum albumin/anionic surfactant SDS [72] and sodium hyaluronate/cationic surfactant tetradecyltrimethylammonium bromide [73]. Here, we first demonstrate this effect for polymer-surfactant complexes with water-insoluble polymer.

Consequently, enhanced screening of the repulsion of headgroups by salt ions in P4VP/potassium oleate complexes not only causes the sphere-to-rod transition of surfactant micelles, but also affects their shape on a large scale. This fact can be confirmed by the corresponding zoomed-in cryo-TEM micrographs (Figure 3).

To sum up, the addition of KCl to the solutions of P4VP/potassium oleate complexes provides the transformation of beads-on-string structures into the short rodlike hybrid micelles. Since hydrophobic P4VP favors the spherical packing of potassium oleate molecules in the micelles, the transformation in polymer-containing solutions occurs at higher concentration of KCl as compared to the polymer-free surfactant solutions. Furthermore, enhanced screening influences the large-scale shape of the complexes, inducing the transition from highly extended to Gaussian coil conformation.

### 3.3. Polymer Inside the Hybrid Micelles of Surfactant-Polymer Complex: Conformation and Localization

**Figure 5:**
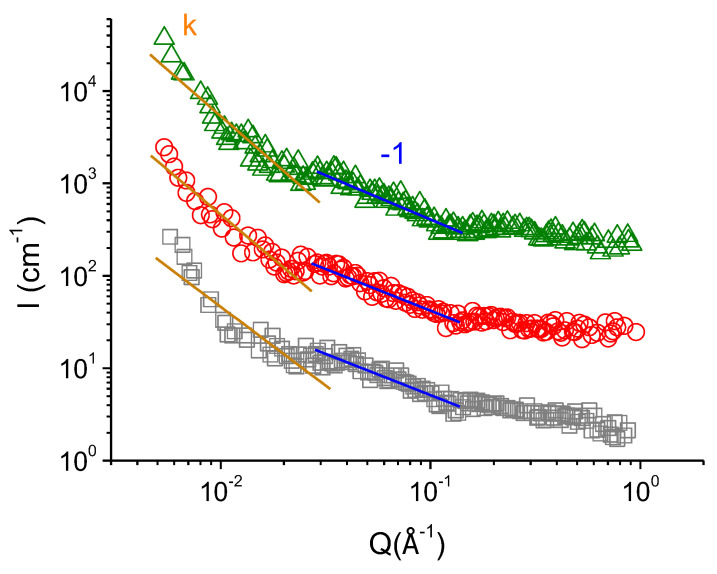
SANS curves of saturated P4VP/potassium oleate complexes in the presence of different concentrations of KCl: 67 mM (squares), 110 mM (circles) and 160 mM (triangles). Solvent: 10 vol% of D_2_O/90 vol% H_2_O at pH 11. Blue solid lines show slopes of the *Q*^−1^ dependences. Orange solid lines show the slope of the curves in the low-*Q* range.

Information about the state of polymer in the P4VP/potassium oleate complexes was obtained by SANS with contrast matching providing the suppression of the scattering from potassium oleate, so that the contribution of polymer to the scattering can be directly studied. It was shown that in the medium-*Q* region, the scattering intensity varies as *I~Q*^−1^. According to the model of persistent polymer chain, the power-law of −1 results from scattering of persistence units [60,67,70]. Low-*Q* region of the curves can be attributed to the scattering from the whole polymer molecule, and the slope *k* in this region (Figure 5) allows judging about the conformation of the polymer chain [67,70,76,77]. The *k* values obtained at different concentrations of KCl are demonstrated in Table 2. It is seen that *k* gradually decreases from −1.7 to −2 with KCl concentration suggesting the change of the conformation of P4VP chain in the complex from the swollen coil to Gaussian coil state [67,70,76,77,78]. It perfectly coincides with the variation of the fractal dimension *d_f_* of P4VP/potassium oleate complexes (Table 1). Consequently, higher screening provokes more compact structures of both polymer and surfactant components of P4VP/potassium oleate complexes.

To estimate the localization of polymer in the polymer/surfactant complex the 2D NOESY technique was used [44,79,80,81]. Note that previously this method was successfully applied to P4VP/potassium oleate complexes in the absence of salt [45]. In that case, pronounced cross-peaks between α- and β-protons of pyridinium ring of P4VP and almost all groups of hydrophobic tail of potassium oleate were observed, which indicated that, in the absence of salt, the polymer occupied the whole micellar interior of the complex [45].

**Figure 6:**
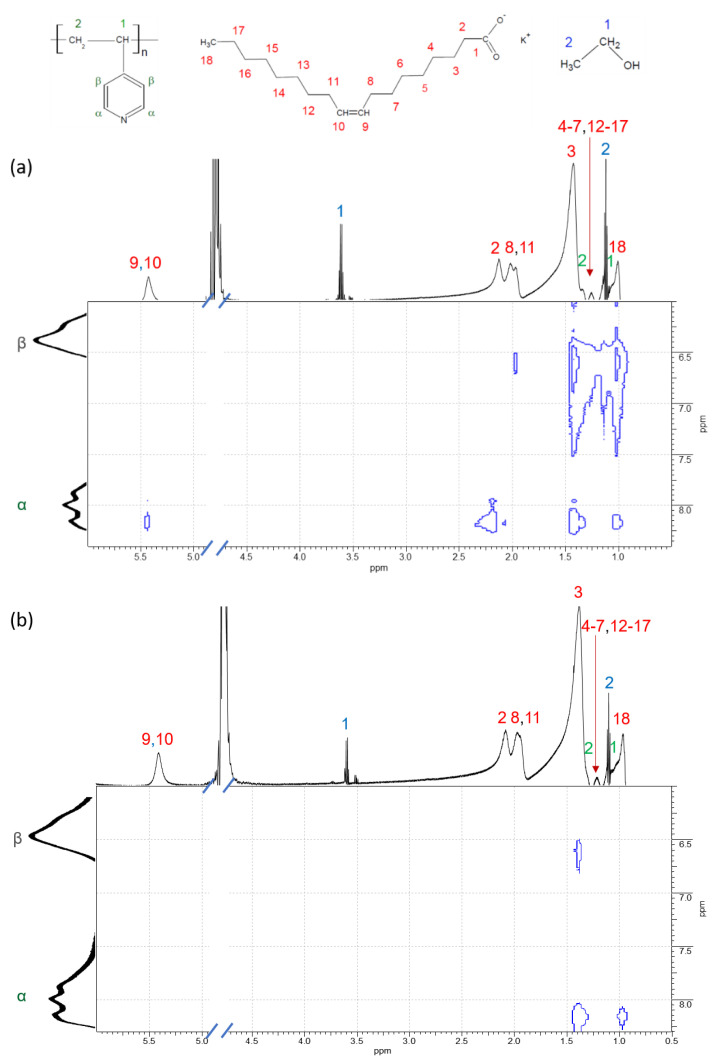
Cross-peaks of the 2D NOESY spectra for the saturated P4VP/potassium oleate complexes in the solutions containing 94 mM potassium oleate and different concentrations of salt KCl: 67 (**a**) and 160 mM (**b**). Solvent: D_2_O at pH = 11; t = 22 °C. The ^1^H NMR peaks assignments of potassium oleate and P4VP were reproduced from reference [65] with permission of Royal Society of Chemistry.

In the present paper, this method was used to reveal the change of the location of P4VP inside the complexes at increasing salt content. The 2D NOESY spectra of the P4VP/potassium oleate solutions containing 67 and 160 mM KCl are depicted in Figure 6a,b, respectively. In 67 mM KCl, α- and β-protons of P4VP strongly interact with most of the methylene (H3, H4-H7, H12-H17) groups of the surfactant tail (Figure 6a). Therefore, at low screening of electrostatic repulsion of surfactant heads by salt the polymer penetrates deep into the micellar core. It is possibly due to mainly spherical packing of surfactant molecules (Figure 3a) which provides more free space for the accommodation of polymer inside the micelles [45]. At the same time, no interaction between the β-protons of P4VP and both double bond and methylene group H2 located most closely to the headgroup of potassium oleate is observed. It indicates that these fragments of the surfactant are not located close to the polymer backbone.

Significant changes of 2D NOESY spectrum are observed at higher salt concentration (cf. Figure 3a,b). In this case, intensive cross-peaks between the pyridinium ring of polymer and most of the methylene groups of the surfactant disappear, except for the one that corresponds to the interaction with the methylene group H3 close to the surfactant head. This observation evidences that P4VP in the hybrid WLMs is located at the interface between the hydrocarbon core and the hydrophilic corona of the micellar aggregates. This result is consistent with atomistic computer modelling demonstrating that P4VP is held at the core-corona interface by the hydrogen bonds between nitrogen in pyridinium ring and water molecules [46]. Such location of P4VP can be explained by insolubility of P4VP both in water and in alkanes (compounds modelling the hydrophobic tail). Therefore, enhanced screening not only affects the form of polymer-containing complexes, but also forces the polymer to be kept in a substantially restricted space, that is, probably, accounts for the conformational transition of P4VP chain from the swollen to Gaussian coil state determined by SANS (Table 2).

**Figure 7:**
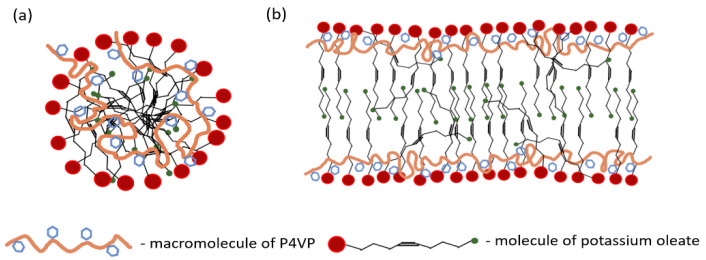
Schematic representation of the structure of the polymer-surfactant complexes formed by P4VP macromolecules (orange and blue) with spherical (**a**) and rodlike (**b**) micelles of potassium oleate (red, black and blue). The alkyl backbone and pyridinium rings of P4VP are shown in orange and blue, respectively. The headgroup, hydrophobic tail and terminal methyl group are shown in red, black and green, respectively.

It is worth to pay attention to the interaction between the methyl group H18 at the end of the hydrophobic tail of potassium oleate and the pyridinium ring of P4VP (Figure 6). Cross-peak of this group with α-protons of the ring is preserved in the spectra at all studied concentrations of KCl, unlike cross-peak with β-protons that disappears at higher concentration (160 mM). The interaction between polymer α-protons and the end-group of the surfactant tail may indicate that the hydrophobic tails of some surfactant molecules inside the micelles are bent (Figure 7). Similar effect was previously assumed by R. Nagarajan for polymer-free spherical and rodlike micelles [8]. The freedom of the terminal methyl group to move inside the micelles was attributed to the non-uniform deformation of the surfactant tail [8].

Thus, an enhanced screening of the P4VP/potassium oleate complexes by salt not only results in the sphere-to-rod transition of the complexes, but also is reflected in the conformational transition and the relocation of the polymer. At low salt concentration, the swollen coil of P4VP occupies almost the whole interior of spherical micelle. At higher salt concentration, P4VP has to acquire more compact Gaussian coil conformation to match a limited space at the core-corona interface of the rodlike micelle (Figure 7b).

## 4. Conclusions

For the first time, the effect of gradual addition of salt on the evolution of the structure of polymer-surfactant hybrid micelles composed of uncharged water-insoluble polymer and ionic surfactant was studied and compared to that of polymer-free micelles. It was shown that at low salt content (67 mM KCl), the hybrid micelles represent beads-on-string structures, where few spherical beads are connected with each other by short polymeric strings. At the same conditions, in polymer-free system, a mixture of spherical and rodlike micelles is formed. It indicates that polymer stabilizes spherical surfactant aggregates (no rods are detected in the hybrid system). At higher salt content (160 mM KCl), an enhanced screening of electrostatic repulsion induces the formation of short rods in the hybrid system and of very long branched WLMs in the polymer-free system. It suggests that semi-spherical end-caps are stabilized by polymer which prevents the growth of micelles in length and their branching. Thus, hydrophobic polymer solubilized within the surfactant micelles shifts the sphere-to-rod transition to higher salt content, makes the rodlike structures shorter and eliminates their branching.

By SANS with contrast variation we are the first to demonstrate that at low salt content, the beads-on-string complexes, containing water-insoluble polymer, are aligned like rigid rods at a scale comparable to the size of the whole macromolecule, probably due to the electrostatic repulsion between similarly charged beads. At higher salt concentrations, the screening of electrostatic repulsion lets the complexes adopt a Gaussian coil conformation. Polymer inside the complexes remains always in the coil state, but increasing salt concentration makes the coils more compact. According to 2D NOESY data, this is accompanied by the relocation of polymer inside the micelles. At low salt content, when the micelles are spherical the polymer penetrates deep into the micellar core, whereas at higher salt content, when the micelles are rodlike, the polymer is localized preferentially at core/corona interface. The obtained results give insight to the salt-responsive behavior of new self-assembled polymer-surfactant complexes based on water-insoluble polymer that are promising as stable smart nanoparticles for various applications including drag reduction.

## Figures and Tables

**Table 1:** Fractal dimensions *d_f_* and characteristic scales *ξ_f_* of the mass-fractal scattering in the solutions of saturated P4VP/potassium oleate complexes in the presence of different concentrations of KCl, obtained upon matching the scattering from P4VP (solvent: 37 vol% D_2_O/63 vol% H_2_O at pH 11).

Concentration of KCl, mM	Fractal Dimension *d_f_*	Characteristic Scale of the Mass-Fractal Scattering *ξ_f_*, nm
67	1.1 ± 0.1	37
110	2.2 ± 0.2	40
160	2.2 ± 0.2	40

**Table 2:** Power-law *k* of the SANS curves in the low-*Q* region for saturated P4VP/potassium oleate complexes in the presence of different concentrations of KCl obtained upon contrast matching of the scattering from potassium oleate (solvent: 10 vol% D_2_O/90 vol% H_2_O at pH 11).

Concentration of KCl, mM	*k*
67	1.7 ± 0.1
110	1.9 ± 0.1
160	2.0 ± 0.1

## Data Availability

The data presented in this study are openly available.

## Supplementary Materials

The following supporting information can be downloaded at: https://www.mdpi.com/article/10.3390/polym14235086/s1. Refs. [45,51,57,65,82,83,84] are cited in Supplementary Materials.
