# Supplementary material for: Salt-Induced Transformations of Hybrid Micelles Formed by Anionic Surfactant and Poly(4-vinylpyridine)
Source: Polymers (Basel). 2022 Nov 23;14(23):5086. doi: 10.3390/polym14235086 (PMC9741239; doi:10.3390/polym14235086)
Supplement: Supplementary file 1 [file polymers-14-05086-s001.zip › polymers-2011253-supplementary.pdf]

Supplementary Materials

for

Salt-induced transformations of hybrid micelles formed by anionic surfactant and poly(4-vinylpyridine)

Alexander L. Kwiatkowski,*[,a] Vyacheslav S. Molchanov,[a] Alexander I. Kuklin,[b] Yuri M. Chesnokov,[c] and Olga E. Philippova[a]

[a] *Physics Department, Moscow State University, 119991 Moscow, Russia*

[b] *Joint Institute for Nuclear Research, 141980 Dubna, Russia*

[c] *National Research Center, Kurchatov Institute, 123182, Moscow, Russia.*

Analysis of decay time distribution functions of polymer-free micelles of potassium oleate and polymer-surfactant complexes with poly(4-vinylpiridine) in 67 mM KCl.

Field autocorrelation functions $g^{(1)}(q,t)$ and the decay time distribution functions *A(t)* of the polymer-free micelles and polymer-surfactant complexes in the presence of 67 mM of potassium chloride are presented in Figure S1. Both autocorrelation functions behave multiexponentially: slow, medium and fast relaxation modes appear for polymer-free micelles and polymer-surfactant complexes.

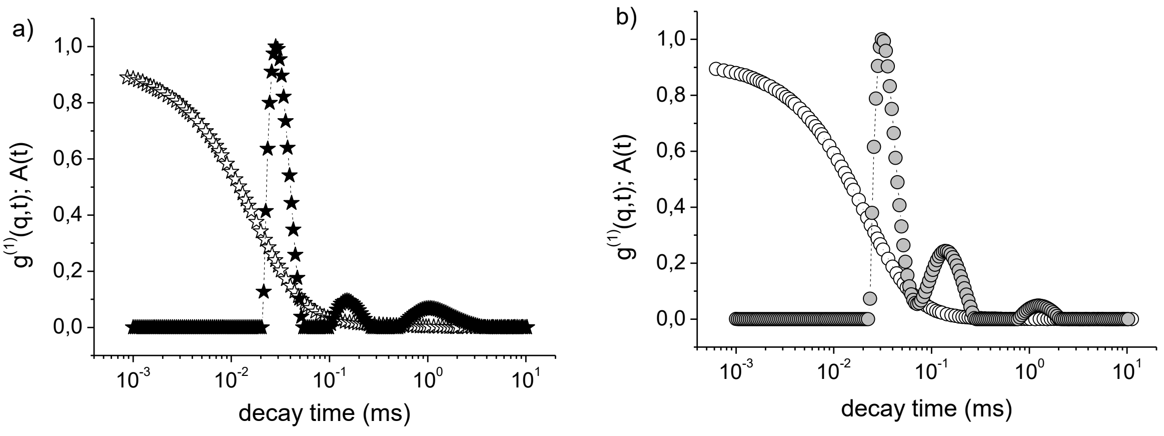

Figure S1. Field autocorrelation function $g^{(1)}(q,t)$ (open symbols) and the decay time distribution function *A(t)* (filled symbols) at scattering angle θ=90° for the aqueous solutions of 94 mM

potassium oleate without polymer (a) and polymer-surfactant complexes, containing 0.14 monomol/L of P4VP (b) in 67 mM KCl.

Dependences of the relaxation rates Γ on the squared scattering vector q^2 determined from the autocorrelation functions $g^{(1)}(q,t)$ are demonstrated in Figures S2 and S3. Linear dependences $\Gamma(q^2)$ for fast and medium modes point out to the diffusion motion of the objects. Corresponding diffusion coefficients D were estimated from the slopes of the dependences. Non-linear dependences for slow relaxation modes $\Gamma_{slow}(q^2)$ indicate that this is a non-diffusive decay. It could be attributed to Coulomb interaction of the charged micelles [2].

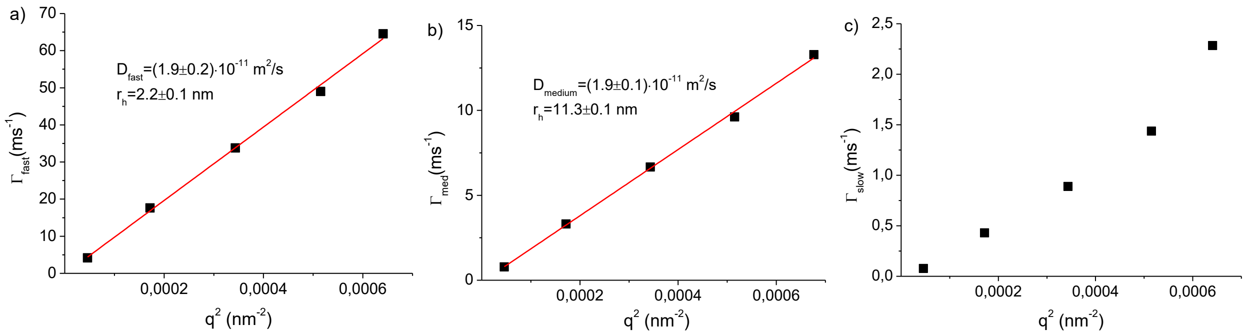

Figure S2. Dependences of the rates Γ of the fast (a); medium (b) and slow (c) relaxation modes on squared values of scattering vector q^2 for aqueous solutions of polymer-free micelles in 94 mM potassium oleate in the presence of 67 mM KCl. Solid lines demonstrate the approximation of the dependences with the straight line.

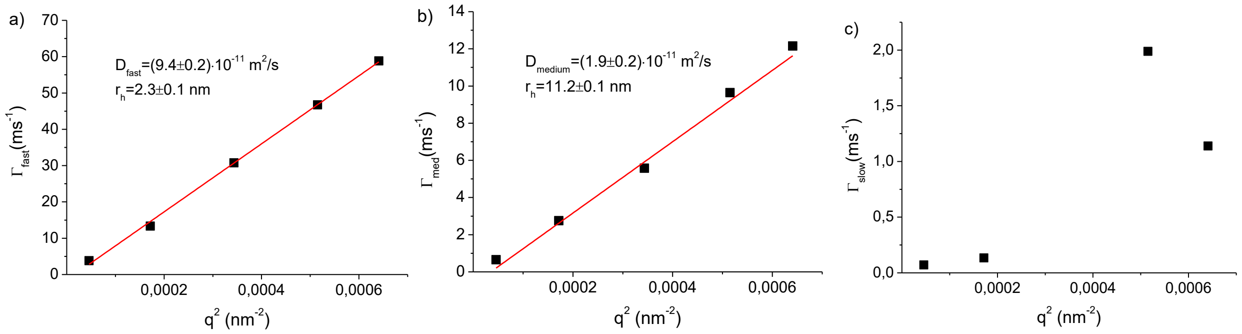

Figure S3. Dependences of the rates Γ of the fast (a); medium (b) and slow (c) relaxation modes on squared values of scattering vector q^2 of aqueous solutions of polymer-surfactant complexes in 94 mM potassium oleate in the presence of 67 mM KCl. Solid lines demonstrate the approximation of the dependences with the straight line.

Using the Stock-Einstein relation, one can obtain the values of the hydrodynamic radii r_h of the effective spheres having the same diffusion coefficients as the scattering objects [1,3]:

$$r_h = \frac{k_B T}{6\pi\eta_0 D}, \quad (1)$$

where k_B is the Boltzman constant, η_0 is the viscosity of the solvent, T is the absolute temperature. The values of r_h are summarized in Table S1.

Table S1. Hydrodynamic radii of the effective spheres r_h, calculated from the corresponding values of the diffusion coefficients D using the Stock-Einstein equation.

<table>
<tr><th rowspan="3">concentration of KCl, mM</th><th colspan="4">r_h, nm</th></tr>
<tr><th colspan="2">fast mode</th><th colspan="2">medium mode</th></tr>
<tr><th>solutions without polymer</th><th>solutions with P4VP</th><th>solutions without polymer</th><th>solutions with P4VP</th></tr>
<tr><td>67</td><td>2.2±0.1</td><td>2.3±0.1</td><td>11.3±0.1</td><td>11.2±0.1</td></tr>
</table>

According to literature [1,3,4], in the solutions of surfactants a fast relaxation mode is due to pure translational diffusion of spherical micelles. The positions of the peak for fast modes in the systems containing P4VP-potassium oleate complexes perfectly coincide with the fast mode observed for polymer-free micelles of potassium oleate (Figure S1). That evidences the similarity of spherical micelles observed in the systems. The values of r_h for spherical micelles in both systems (Table S1) correlate with the length of hydrophobic tail of potassium oleate [5].

As to the medium mode, it is much more pronounced in polymer-containing system (Figure S1). In the polymer-free solution, the possible origin of the medium mode is the translational diffusion of the short rodlike micelles of potassium oleate, which are seen on the cryo-TEM picture (Figure 1a). From their value of r_h (Table 1), the length of micelles L could be evaluated from the following relation for hydrodynamic radius of rodlike object:[3]

$$r_h = \frac{L}{\ln\left(L/d\right)^2 + 0.62 + 1.13\, d/L}, \quad (2)$$

where d is the diameter of the micelles equal to 3.8 nm in the case of potassium oleate [5]. The estimation from (2) gives the length of micelles equal to ca. 80 nm. It corresponds to the length of rodlike micelles observed in cryo-TEM image (Figure 1a).

To shed light on the origin of the medium mode of polymer-surfactant complexes, one has to evaluate the value of hydrodynamic radius of beads-on-string structure representing the spherical micelles with r_h=2.3 nm attached to P4VP chain. According to SANS, in 67 mM KCl

the polymer in the complexes is in the swollen coil conformation (Table 2). The following expression should be used to obtain the r_h of polymer [92]:

$$r_h = \left(\frac{3\pi}{128}\right)^{1/2} \left(2L_{P4VP} l_p\right)^{3/5}, \tag{3}$$

where L_{P4VP} and l_p are the contour and the persistent lengths of P4VP equal to 183 and 0.8 nm [6], respectively. From the expression (3) r_h of P4VP can be estimated as 8.2 nm. Consequently, r_h of the necklace, containing spherical beads, equals to ca. 10.5 nm. This value is close to the value determined by DLS (Table S1). Therefore, the medium relaxation mode in the solutions of beads-on-string polymer-surfactant complexes is caused by their translational diffusion.

Thus, the DLS data coincide with cryo-TEM visualization results.

References:

[1] E.V. Korchagina, O.E. Philippova, Multichain aggregates in dilute solutions of associating polyelectrolyte keeping a constant size at the increase in the chain length of individual macromolecules, Biomacromolecules. 11 (2010) 3457–3466. https://doi.org/10.1021/bm100990u.

[2] J. Li, T. Ngai, C. Wu, The slow relaxation mode: From solutions to gel networks, Polym. J. 42 (2010) 609–625. https://doi.org/10.1038/pj.2010.59.

[3] P.A. Hassan, S. Rana, G. Verma, Making sense of brownian motion: Colloid characterization by dynamic light scattering, Langmuir. 31 (2015) 3–12. https://doi.org/10.1021/la501789z.

[4] S. Chodankar, V.K. Aswal, P.A. Hassan, A.G. Wagh, Structure of protein-surfactant complexes as studied by small-angle neutron scattering and dynamic light scattering, Phys. B. 398 (2007) 112–117. https://doi.org/10.1016/j.physb.2007.05.003.

[5] V.S. Molchanov, O.E. Philippova, A.R. Khokhlov, Y.A. Kovalev, A.I. Kuklin, Self-assembled networks highly responsive to hydrocarbons, Langmuir. 23 (2007) 105–111. https://doi.org/10.1021/la061612l.

[6] A.L. Kwiatkowski, V.S. Molchanov, H. Sharma, A.I. Kuklin, E.E. Dormidontova, O.E. Philippova, Growth of wormlike micelles of surfactant induced by embedded polymer: role of polymer chain length, Soft Matter. 14 (2018) 4792–4804. https://doi.org/10.1039/C8SM00776D.

[7] A.L. Kwiatkowski, V.S. Molchanov, A.I. Kuklin, A.S. Orekhov, N.A. Arkharova, O.E. Philippova, Structural transformations of charged spherical surfactant micelles upon solubilization of water-insoluble polymer chains in salt-free aqueous solutions, J. Mol. Liq. 347 (2022). https://doi.org/10.1016/j.molliq.2021.118326.
